# LPG–PCFG: An Improved Probabilistic Context- Free Grammar to Hit Low-Probability Passwords

**DOI:** 10.3390/s22124604

**Published:** 2022-06-18

**Authors:** Xiaozhou Guo, Kaijun Tan, Yi Liu, Min Jin, Huaxiang Lu

**Affiliations:** 1 Institute of Semiconductors, Chinese Academy of Sciences, Beijing 100083, China; xiaozhouguo@foxmail.com (X.G.); tkj@semi.ac.cn (K.T.); liuyi@semi.ac.cn (Y.L.); luhx@semi.ac.cn (H.L.); 2 University of Chinese Academy of Sciences, Beijing 100089, China; 3 Materials and Optoelectronics Research Center, University of Chinese Academy of Sciences, Beijing 200031, China; 4 College of Microelectronics, University of Chinese Academy of Sciences, Beijing 100049, China; 5 Semiconductor Neural Network Intelligent Perception and Computing Technology Beijing Key Laboratory, Beijing 100083, China

**Keywords:** information security, password-generation model, PCFG, low-probability password, degeneration distribution

## Abstract

With the development of the Internet, information security has attracted more attention. Identity authentication based on password authentication is the first line of defense; however, the password-generation model is widely used in offline password attacks and password strength evaluation. In real attack scenarios, high-probability passwords are easy to enumerate; extremely low-probability passwords usually lack semantic structure and, so, are tough to crack by applying statistical laws in machine learning models, but these passwords with lower probability have a large search space and certain semantic information. Improving the low-probability password hit rate in this interval is of great significance for improving the efficiency of offline attacks. However, obtaining a low-probability password is difficult under the current password-generation model. To solve this problem, we propose a low-probability generator–probabilistic context-free grammar (LPG–PCFG) based on PCFG. LPG–PCFG directionally increases the probability of low-probability passwords in the models’ distribution, which is designed to obtain a degeneration distribution that is friendly for generating low-probability passwords. By using the control variable method to fine-tune the degeneration of LPG–PCFG, we obtained the optimal combination of degeneration parameters. Compared with the non-degeneration PCFG model, LPG–PCFG generates a larger number of hits. When generating $10^{7}$ and $10^{8}$ times, the number of hits to low-probability passwords increases by 50.4% and 42.0%, respectively.

## 1. Introduction

Since the birth of computers in the last century, the use of passwords has become a widespread way to verify a user’s identity [1]. This system is simple to program and easy to use, which means the password authentication system could exist for a long time [2]. To meet safety concerns, a good password must be long and irregular. However, in practice, people have tens or hundreds of accounts to manage, so [3] saving passwords in a notebook or software might also cause a leakage risk [4,5]. Later, people used human-memorable passwords, which can be attacked [6,7]. We usually measure the strength of a password using the concept of guessing time. If a password is set using ten small letters, the attacker needs to try at most $26^{10}$ guesses.

Businesses, especially small and medium enterprises, suffer from cyber-breaches [8], and not fixing them in time can lead to data breaches [9]. If the victim uses plain text to save passwords, attackers can use the leaked username–password pair to stuff the credentials of users onto other service providers [10], which seriously damages the users’ information security. To protect user passwords, a website usually saves the calculation result of one-way hash functions such as SHA256 in the database rather than the plain text, which significantly increases the cost of attacks [11,12]. For higher protection, the websites can add a random string into plain passwords and calculate the hash value of the processed string, called the “salted hash” [13]. It has been mathematically proven that, for a given hash function such as SHA256, calculating the original text back from a given hash value is almost impossible [14]. However, calculating the hash value of a given text is relatively easy, so the attacker’s only means of obtaining the password from the hash value is to guess each password and verify its hash value. Attackers can save all the hash values of each password they have ever tried, and they would crack the target hash value when it meets the exact hash text [15]. Rainbow tables can accelerate this process using the time–space trade-off method [16]. Recovering passwords from hash values is usually called an offline attack.

It is hard to hit a hash value calculated by a long random string for the salted hash [17]. To accelerate the attack speed, some researchers focus on improving the hash calculation speed to make attackers try faster [18], including designing a special application-specific integrated circuit (ASIC) to calculate hash functions and deploy the program on distributed computers [19,20,21,22]. Accelerating the guessing speed is crucial to making more guesses, but our research in this article focuses on generating high-quality passwords. As long as people keep using human-memorable passwords, most of them will have patterns and laws [23], in which case the guessing time for the attacker could be much shorter than the worst-case scenario: $s^{l}$, where *s* is the size of all possible characters and *l* is the maximum length of the password [24]. The password-generation model based on machine learning can mine password data to generate good passwords in large quantities [25]. For this reason, it has become a viable option for efficient password cracking.

There are different ways to build a password-generation model. A directed method is the “brute force attack”, which entails trying all possible combinations one by one [26]. Another method is the “dictionary attack” in which a list of all possible passwords is tried one by one [27,28]. These were quite useful for early systems that had small password length limits. With the development of information systems, password-generation methods based on password dictionaries and heuristic rules [29] were proposed and became the popular methods for guessing a password. Later, a password-generation model based on the Markov process appeared [16]. Then came probabilistic context-free grammar (PCFG) [30], which can automatically learn from training data. With the development of deep learning, password-generation models based on an artificial neural network (ANN) have also been proposed, such as long short-term memory (LSTM) [31] and generative adversarial network (GAN) [32].

During an offline password attack, high-probability passwords such as 123456 are limited and easy to cover by a trained password-generation model. Completely random passwords with an extremely low probability, such as 2jca3*4, do not have semantic information; therefore, we should not use machine learning models to generate them. However, low-probability passwords such as amen-1999-01 still contain obvious semantic information and have large numbers [33]. In this work, the focus is on improving machine learning models to improve the generation of low-probability passwords. On the one hand, more low-probability passwords will provide attackers with better password dictionaries in offline attacks. On the other hand, the strength of the passwords can be evaluated better by low-probability models to protect user information.

Our contributions are as follows:

1. We propose a degenerate distribution algorithm suitable for machine-learning-based password-generation models to generate low-probability passwords effectively.
2. We apply the algorithm to the PCFG model, which significantly improves the low-probability password hit numbers.
3. We explore the improvement in low-probability password generation of the model when applying the degenerate algorithms using different parameters to different parts of PCFG.

The structure of this paper is as follows. In Section 2, we summarize different kinds of guessing methods. In Section 3, we introduce the low-probability generation– probabilistic context-free grammar (LPG–PCFG) model based on the degeneration distribution. In Section 4, we show our experiment result and analyze the reason for the difference in each parameter. In the last section, we present our conclusion and prospects for the future research.

## 2. Related Work

Password-attacking algorithms can be divided into brute force and dictionary cracking. In the brute force cracking method, attackers try to exhaust all strings that satisfy a particular requirement through an enumeration algorithm [34]. With the help of a graphics processing units (GPUs) and distributed computing [35], this method may be efficient in a small password space. These two attacks were first proposed to attack the UNIX security system [36]. However, when the maximum length of the password and the size of the character dictionary increase, the number of operations increases exponentially. Currently, it is usually difficult to traverse all strings with limited computing resources [37]. In the dictionary-cracking method [38], attackers first generate a dictionary containing a large number of potential passwords and then try to crack the password. To increase the crack rate, some passwords in the dictionary are transformed by setting rules. Hashcat [39] sets some common transformation rules to simulate human password creation, for example by turning “love” into “l0ve”. The program John the Ripper [40] modifies, cuts, and expands words and adds more rules, so it could be more flexible and efficient in cracking.

These two methods are not effective, especially in a huge password space, because they have to perform a huge number of attempts, and some generated passwords are almost meaningless [41]. Instead of attempting a traversal search, generation methods based on machine learning directly learn the probability distribution of passwords, so they obtain better results.

### 2.1. PCFG

The PCFG [30] model splits a password into several variables according to the type of character (letter, digit, or special), so it can model the characters of different parts separately. Houshmand adds keyboard rules in PCFG to consider the relationship of adjacent characters on the keyboard [42]. This rule makes the model crack some extra passwords that follow keyboard rules. Vears performs deep semantic mining on the letter variable [43]. For passwords, it performs segmentation and parts-of-speech tagging operations. The model replaces a letter with a similar word on the basis of semantic analysis. Li proposes a Personal-PCFG that treats the user name, email prefix, name, birthday, mobile phone number, and ID card as new variables [44] that have the same status and positively affect targeted attacks. In addition, other common content such as Chinese Pinyin, date, and common character combinations have also been added to the PCFG model [45], which inspired Deng to construct a conditional random field generation model [25]. Han realized the syntax knowledge transfer from short to long passwords by using a transPCFG model.

On the whole, compared with other models based on statistical learning, the PCFG model has finer modeling granularity [46]. It subdivides the high-probability password structure to increase the number of passwords generated and improve the cracking ratio. Experiments show that it can crack more passwords because of the finer structural divisions.

### 2.2. Other Password-Generation Models

Other models are based on different assumptions, but they also achieve good cracking. The Markov model thinks that passwords just have local relevance so that each character just correlates with its first several characters [16]. The Markov model treats all characters equally regardless of type. Tansey proposes a multilayer Markov model that expands the number of layers from one to *n*. More layers give the model a stronger representational ability to generate better passwords [47]. Based on the Markov model, Durmuth introduced OMEN, which generates passwords in descending order of probability [48]. OMEN makes repeating a password impossible, so it greatly improves cracking efficiency. Guo proposes a dynamic Markov model, which reduces the repetition rate of password generation [49]. Experimental results show that it definitely has advantages in a targeted attack. The Markov model and its variants usually have a good comprehensive performance [50].

A neural network with deep layers usually has a larger capacity and better feature extraction capability, so the password-guessing models based on deep learning have received more attention [51]. Sutskever first generated long text using a recurrent neural network (RNN), which indicated that it was suitable for capturing the relationship between characters [52]. Since a password is essentially a sequence of strings, Melicher used an RNN to generate passwords [31]. The RNN outputs a character in each time step and receives it as the input of the next time step. Xu improved the network architecture and replaced the RNN with LSTM, to mine long-range dependency [53]. Teng proposes PG-RNN, which increases the number of neurons and has a competitive effect on different datasets [54].

The GAN [55] is a powerful generation model that has a strong learning ability in computer vision [56,57] and natural language processing [58]. Hitaj introduces PassGAN to generate passwords [32]. In the PassGAN model, every character is encoded by a one-hot vector and the password is organized into a sparse matrix. The model implicitly learns the probability distribution of the password by minimizing the distance between a fake and real distribution. Nam uses relative GAN to improve the objective function, and it greatly improves password generation through multisource training [59]. Nam improves the generator by using an RNN to obtain a better iterative representation [60]. Guo analyzes the generation effect of the GAN and proposes a PG-GAN model that can reduce the password repetition rate [61].

First, compared to the RNN and GAN models, PCFG has an apparent speed advantage. During training, many weight parameters have to be trained, and some problems such as “non-convergence mode collapse” may appear [62]. In training, PCFG just needs to count frequency, but in the generation process, it needs fewer calculations than a neural network, which has to perform a large number of multiplication and activation operations. Then, considering password generation quality, the assumption of the Markov model is simple and the learning ability of the neural network is restricted to model capacity [63]. Passwords generated by PCFG usually comply with most password patterns, and mining semantic information to letter variables could guarantee a reasonable password. Eventually, the neural network model generates duplicate passwords, especially in GAN.

In summary, the PCFG model has fine-grained modeling accuracy and has an advantage in comprehensive ability, including the time cost and quality of generated passwords. Therefore, we modified the PCFG model to generate low-probability passwords.

## 3. Method

### 3.1. Random Sampling

**Figure 1:**
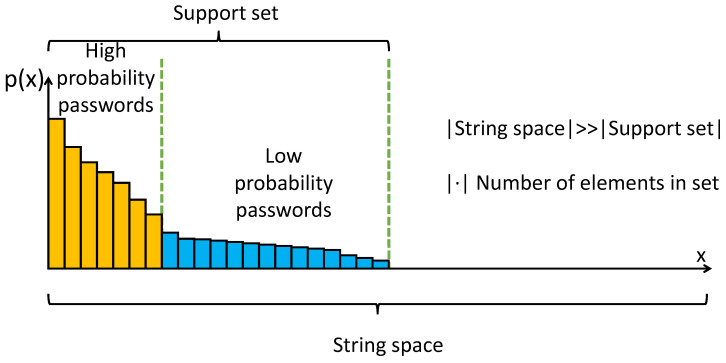
Relationship among string space, support set, and low-probability password.

After completing the training of the password-generation model *G* using the training dataset, a probability value is assigned to each password *x* on the support set $S_{M}$, as shown in Figure 1. Since the password distribution approximately conforms to the Zipf law [64,65], the frequency of a password is inversely proportional to its frequency ranking in the password set. There will be many low-probability passwords in the password distribution P(x), but it will be tough to use enumeration and random sampling directly. Next, we analyze these two methods.

For the generation model based on enumeration, the password is usually generated in approximate descending order of probability; that is, the passwords with high probability are first generated, and then, the passwords in a lower probability interval are generated. Therefore, in the early stage of inferencing of the model, many passwords that are not in the low-probability interval will be generated. Regarding the design of the search algorithm, the enumeration method inevitably traverses and accesses high-probability passwords when searching for low-probability password intervals, which cannot be the model’s sole focus because that would be a waste of computing resources. Finally, the range of low-probability password intervals is extensive compared to those of high-probability, and searching for low-probability passwords would result in unacceptable computational overhead.

The password-generation model can be regarded as random sampling directly from the probability distribution P(x). However, since the chance of occurrence is related to its probability value, the model usually prefers high-probability passwords and has a weaker preference for generating low-probability passwords. Considering the inhomogeneous distribution of password probability values in P(x), there is a magnitude difference between the values of high-probability and low-probability passwords, so directly using random-sampling-based methods to generate low-probability passwords will have lower efficiency.

Compared to the random generation method, the password-generation model based on random sampling reduces the attention range from the string space $S_{S}$ to the support set $S_{M}$ so that the learned password features can be used fully, leading to a more extensive, more significant low-probability password-generation potential. Compared to the enumeration method, random sampling does not establish a mandatory password output priority, and in any random sampling, a password with any probability may be generated. Although the frequency of passwords follows a statistical law in a random sample, this method retains many possibilities for creating low-probability passwords. We chose to optimize the password-generation model based on random sampling to adapt it to low-probability tasks.

### 3.2. Degeneration Distribution

**Figure 2:**
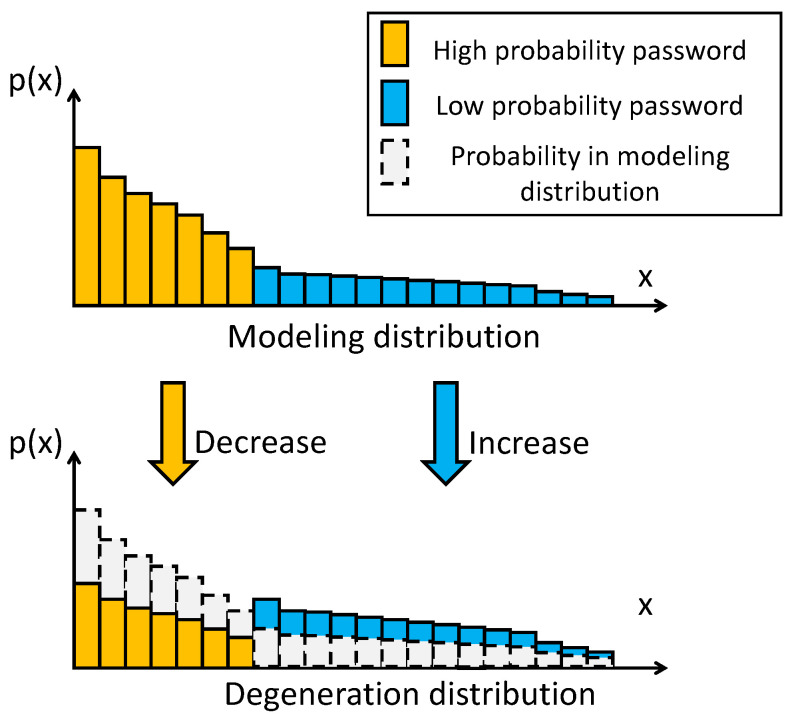
Evolution relationship between modeling distribution and degeneration distribution.

The trained password-generation model models the password, and its distribution is recorded as the distribution $D_{ori}$. In $D_{ori}$, the probability value of the low-probability password $x_{l}$ relative to the high-probability password $x_{h}$ has many orders of magnitude difference. The degeneration distribution $D_{deg}$ is a password distribution obtained from the evolution of the modeling distribution $D_{ori}$. The difference between the probability values of $x_{l}$ and $x_{h}$ is significantly reduced, as shown in Figure 2. Random sampling from the degenerate distribution $D_{deg}$ can improve the generation of low-probability passwords.

The degeneration distribution $D_{deg}$ is an intermediate state between the modeling and uniform distributions $D_{uni}$, where $D_{ori}$ retains all the learned password features. At the same time, $D_{uni}$ cannot reflect any password features; it assigns the same probability value to all passwords in the support set. In its initial evolution from the modeling distribution $D_{ori}$ to $D_{uni}$, the closer the degeneration distribution comes to the uniform distribution, the better the distribution will be for generating low-probability passwords. However, the generated passwords will lack the learned features when the degeneration distribution is very close to the uniform distribution. It is challenging to trade-off high-quality and low-probability passwords in a limited number of generation times. In summary, there is an optimal degeneration distribution $D_{deg}^{*}$ that can achieve a balance between the modeling and uniform distributions so that many low-probability passwords can be generated efficiently.

To obtain the degeneration distribution, a high-probability password in the modeling distribution $D_{ori}$ is necessary; then, it is possible to modify its probability value. The random sampling method has a natural preference for high-probability passwords, which means they can be extracted by random sampling in $D_{ori}$, after which the degeneration distribution can be obtained by directly reducing the probability value. To make the degeneration distribution always take the uniform distribution as the endpoint in the evolution, we adopted the following mechanism: whenever the password $x^{+}$ was obtained by sampling the generation model, its probability was modified to $p(x^{+})-\alpha$, and the $x^{-}$ possibility of other passwords in the support set was changed to $p\left(x^{-}\right)+\alpha /\left(N_{S}-1\right)$, where $N_{S}$ represents the number of password elements in the support set. This was recorded as Rule 1, as shown in Table 1. In addition, considering that the granularity of PCFG modeling is small, we designed three different probability modification rules, denoted as Rules 2–5, to find the optimal degeneration distribution. The specific methods are shown in Table 1.

Next, we explain that the five password probability adjustment rules in Table 1 can change the degeneration distribution approach in the direction of a uniform distribution. We used the Kullback–Leibler (KL) divergence to measure the distance between the degenerate $D_{deg}$ and uniform distributions $D_{uni}$:

(1) $$D_{KL}(D_{uni}\left|\right|D_{deg})=-logN_{S}-\frac{1}{N_{S}}\sum_{i=1}^{N_{S}}log\;p_{i}^{deg}$$

where $p_{i}^{deg}$ represents the probability of password $x_{i}$ in the degenerate distribution. We performed a first-order Taylor expansion in the neighborhood of $p^{deg}=\left[p_{1}^{deg},p_{2}^{deg},\cdots ,p_{N}^{deg}\right]$, and this approximate expression was obtained:

(2) $$D_{KL}(D_{uni}\left|\right|D_{deg})\approx -log\;N_{S}-\frac{1}{N_{S}}\sum_{i=1}^{N_{S}}log\;p_{i}^{deg}-\frac{1}{N}\sum_{N}^{i=1}\frac{1}{p_{i}^{deg}}\left(p_{i}^{new}-p_{i}^{ori}\right)$$

The distribution after the small adjustment of $D_{deg}$ is denoted as $D^{new}$, and the corresponding password probability is $p^{new}=\left[p_{1}^{new},p_{2}^{new},\cdots ,p_{N}^{new}\right]$. We denote the probability of the currently generated password $x^{+}$ as $p_{i}^{new}$ and all other passwords $x^{-}$ as $p_{j}^{new}$ with $j\in 1,2,\ldots ,N_{S}$. To compare the changes in KL divergence caused by the above rule adjustment, we had to verify the positive and negative first-order term A of the approximate expression of the KL divergence. In Rule 1, $p_{i}^{new}=p_{i}^{ori}-\alpha$ and $p_{j}^{new}=p_{j}^{ori}+\alpha /\left(N_{S}-1\right)$, so *A* becomes

(3) $$A=\frac{1}{N}\frac{\alpha }{p_{i}^{ori}}-\frac{1}{N}\sum_{j\in \{1,2,\cdots ,N\}}\frac{1}{p_{i}^{ori}}\frac{\alpha }{N-1}$$

According to the inequality,

(4) $$\frac{1}{x_{1}}+\frac{1}{x_{2}}+\cdot \cdot \cdot +\frac{1}{x_{n}}\geq \frac{n^{2}}{x_{1}+x_{2}+\cdots +x_{n}}s$$

We can obtain:

(5) $$A\leq \frac{1}{N}\frac{\alpha }{p_{i}^{ori}}-\frac{1}{N}\frac{\alpha }{N-1}\frac{{\left(N-1\right)}^{2}}{1-p_{i}^{ori}}$$

When $p_{i}^{ori}\times N\leq 1$, A≤0. In Rule 2, $p_{i}^{new}=p_{i}^{ori}-\alpha$ and $p_{j}^{new}=p_{j}^{ori}+\alpha /\left(1-p_{i}^{ori}\right)p_{j}^{ori}$, so then, *A* becomes

(6) $$A=\frac{1}{N}\frac{\alpha }{p_{i}^{ori}}-\frac{1}{N}\sum_{j\in \{1,2,\ldots ,N\}}\frac{\alpha }{1-p_{i}^{ori}}$$

Obviously, if $p_{i}^{ori}\times N\geq 1$, then A≤0. In Rule 3, $p_{i}^{new}=\beta p_{i}^{ori}$ and $p_{j}^{new}=p_{j}^{ori}+\left(1-\beta \right)p_{i}^{ori}/\left(N-1\right)$, so then, *A* becomes

(7) $$A=\frac{1}{N}\left(\beta -1\right)-\frac{1}{N}\sum_{j\in \{1,2,\ldots ,N\}}\frac{1}{p_{j}^{ori}}\frac{\left(1-\beta \right)p_{i}^{ori}}{N-1}$$

According to the inequality, we have

(8) $$A\leq \frac{1}{N}\left(\beta -1\right)-\frac{N-1}{N}\left(1-\beta \right)p_{i}^{ori}$$

If $p_{i}^{ori}\times N\geq 1$, then A≤0. Finally, in Rule 5, $p_{i}^{new}=1-\left(1-p_{i}^{ori}\right)\gamma$ and $p_{j}^{new}=\gamma p_{j}^{ori}$, so then, *A* becomes  

(9) $$A=-\frac{1}{N}\frac{1-\left(1-p_{i}^{ori}\right)\gamma -p_{i}^{ori}}{p_{i}^{ori}}-\frac{N-1}{N}\left(\gamma -1\right)$$

Similarly, if $p_{i}^{ori}\times N\geq 1$ is satisfied, then A≤0. It can be shown that when the probability of model *G* generating a password is above the average value and the evolution of the degenerate distribution is performed according to Table 1, the degenerate distribution will continue to move closer to a uniform distribution.

### 3.3. LPG–PCFG

**Figure 3:**
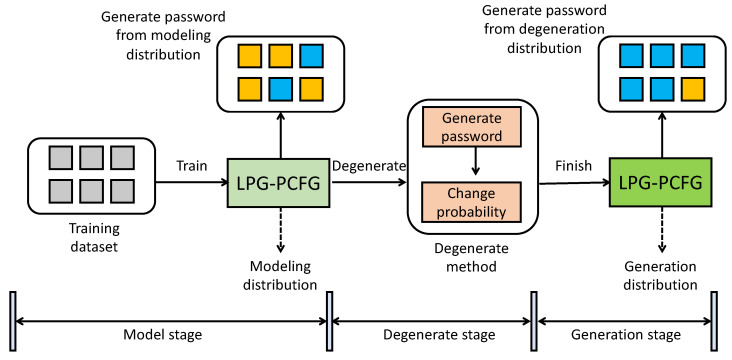
Evolution relationship between the modeling and degeneration distributions.

We applied the degeneration distribution acquisition method described above to the PCFG model and designed the corresponding LPG–PCFG model, for which training was divided into two stages: modeling and degeneration. In the modeling stage, LPG–PCFG learned to obtain the modeling distribution $D_{ori}$ through the training dataset; in the degeneration stage, the model mainly learned the degeneration distribution $D_{deg}$. In the inference phase, we used the random sampling algorithm to sample $D_{deg}$ for password generation, as shown in Figure 3.

#### 3.3.1. Modeling Stage

The LPG–PCFG model distinguishes the characters in the password into three types: letters (case-insensitive) *l*, numbers *d*, and characters *s*. The letter part contains 26 letters, the number part 10 numbers, and the character part symbols such as !, @, …, ?. The structure of any password can be parsed from left to right according to its character type. For example, the corresponding structure of the password 123abc123!! is $d_{3}l_{3}d_{3}s_{2}$. We denote $l_{3},d_{3},s_{2}$ as letter, numeric, and special character variables. The LPG–PCFG model assumes that different variables are independent of each other, so the probability of a password is the product of the structural probability and each partial probability. For example, the probability of 123abc!! is

(10) $$p\left(123abc!!\right)=p\left(d_{3}l_{3}s_{2}\right)p\left(123|d_{3}\right)p\left(abc|l_{3}\right)p\left(!!|s_{2}\right)$$

**Figure 4:**
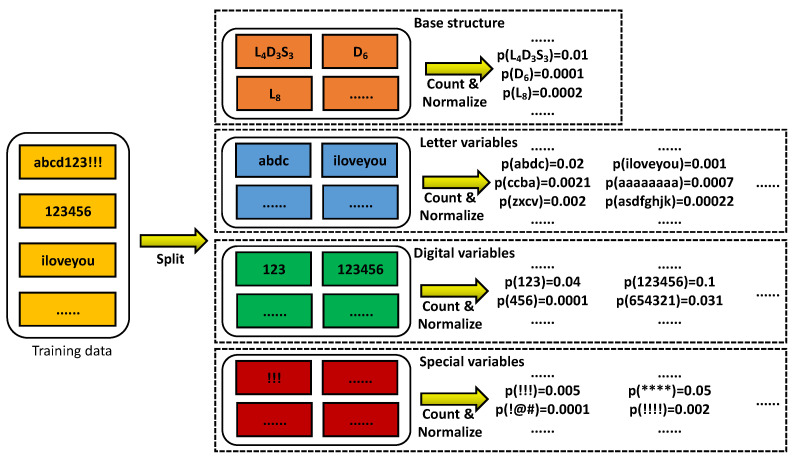
Schematic diagram of the LPG–PCFG model training process.

We parsed the password structure for each password in the training dataset and decomposed it into several parts of letters, numbers, and special characters during the modeling stage. For numeric variables, we counted the occurrence frequencies of those of different lengths, such as $d_{1}$, $d_{2}$, $d_{3}$. For special character and letter variables, it was necessary to conduct similar frequency statistics. All password structures were considered uniformly, and the counts are the different frequency of occurrence of the structure. By normalizing the results of the structure statistics ps and $d_{1},d_{2},d_{3}\ldots s_{1},s_{2},s_{3}\ldots l_{1},l_{2},l_{3}\ldots$ and the statistical results of different length variables, we obtained the probability distribution $D_{ps}$ of the password structure and the probability distributions of different variable types as $D_{d_{1}},D_{d_{2}},D_{d_{3}}\ldots D_{s_{1}},D_{s_{2}},D_{s_{3}}\ldots D_{l_{1}},D_{l_{2}},D_{l_{3}}$, as shown in Figure 4.

The letter part usually contains much complex semantic information, so directly treating it as the whole string will not achieve fine-grained modeling. The LPG–PCFG model uses an n-gram language model for further semantic mining to model the letter parts. Each letter in the section has only a probabilistic connection to its *n* preceding letters, but not to the other letters. We denote $c_{n}|c_{1},c_{2},\ldots ,c_{n-1}$ as n-gram segments, where $c_{i}$ represents a particular letter. A letter variable $l_{m}$ containing m letters can be decomposed into *m* n-gram segments, so the probability calculation method of the letter variable $l_{m}$ is the probability multiplication of multiple n-gram segments. For example, the probability of the letter string abc is p(a)p(b|a)p(c|ab).

#### 3.3.2. Generation Stage

Since the password needs to be generated in the LPG–PCFG model in both the degeneration and generation stages, we first describe the generation method of the LPG–PCFG model.

**Figure 5:**
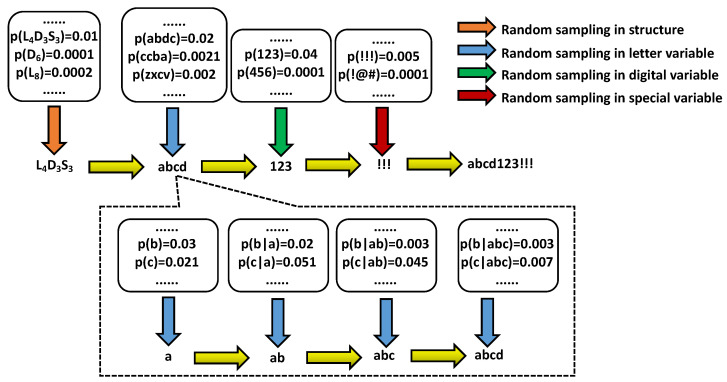
Schematic diagram of LPG–PCFG model generation process.

When generating a password, the password structure probability distribution $D_{ps}$ must first be randomly sampled to obtain a specific structure, for example, the selected structure $l_{4}d_{3}s_{3}$. Then, the variables of different parts are filled in independently from left to right. The digital $d_{n}$ and the special character variables $s_{n}$ can be directly obtained by random sampling in the probability distribution $D_{d_{n}}$ and $D_{s_{n}}$. For the the letter variables $l_{n}$, it is necessary to sample continuously n times according to the n-gram model to obtain *n* letters, that is, continuous sampling in the conditional probability distributions such as $p\left(C\right),p\left(C|c_{1}\right),p\left(C|c_{1}c_{2}\right),\ldots$. The password-generation process is shown in Figure 5.

#### 3.3.3. Degeneration Stage

In the degeneration stage, whenever a password $x^{+}$ is generated, its probability value $p(x^{+})$ needs to be reduced. When applying the probability adjustment idea to the LPG–PCFG model, we considered the following four aspects:

(1) The probability expression of a password is composed of four probability factors: structural, numerical, special character, and letter n-gram. If the probability is reduced for all factor parts during adjustment, the password probability value having the same character part as the password will also be significantly reduced, which may cause some low-probability passwords to disappear directly. For example, the selected high-probability password “abcd” is very easy to obtain by sampling, while the password “abcd!12” has a low probability. If p(a),p(b|a),p(c|ab), and p(d|abc) are reduced at the same time, the probability of the password ” may be reduced to such an extent that it is a challenge to be sampled later. Therefore, the LPG–PCFG model selects only one factor for probabilistic modification.

(2) When modifying the probability of the factor, the conditional probability distribution of the letter part, which includes up to 26 elements, is relatively simple. The distributions $D_{s_{n}}$ may include many elements, especially when n is relatively large, even into the thousands or tens of thousands. In addition, the password structure distribution $D_{ps}$ also includes more elements. The high-frequency adjustment of these probability distributions with many features requires a sizeable computational resource overhead, and this limitation also made us choose only one factor for probability modification.

(3) When we adjust the probability of a single factor, we only reduce it to a certain extent, not to 0. As shown in the previous example, if the probability of p(b|a) is directly reduced to 0, the likelihood of the password “abcd!12*” also becomes 0 correspondingly, due to the multiplicative relationship of conditional probabilities. If p(b|a) is not modified afterward, the likelihood of “abcd!12*” always remains 0, so it is impossible to sample.

(4) For the modeling distribution of the LPG–PCFG model, the size of its support set $|S_{M}|$ is much smaller than that of the string space $|S_{S}|$. For the password that has a probability assigned as 0 by the LPG–PCFG model, its feature is fragile. The passwords in the support set and the string space may have shared strings, but to ensure that the probability adjustment does not affect the non-support set part in the string space, the LPG–PCFG model only restricts adjustment of the probability distribution to the support set. The other passwords in the area always have a probability value of 0.

To sum up, the degeneration distribution used by the LPG–PCFG model is as follows: When the password $x^{+}$ is generated, a distribution P(C) is randomly selected from the distribution of the password structure, numeric variable, special character variable, and n-gram conditional probability distribution in the letter variable, where *C* is a random variable and the distribution values in *C* are $c_{1},c_{2},c_{3}\cdots$, while the corresponding probability is $p\left(c_{1}\right),p\left(c_{2}\right),p\left(c_{3}\right)\ldots$. Let the element that increases the probability be $c_{i}$ and the element that decreases the probability be $\left(c_{j}\right)$, where j∈{1,2,…}. The LPG–PCFG model correspondingly designs the modification method of P(C) according to the above five rules. (1) Subtracting the constant α from $p(c_{i})$ gives $p(c_{i})-\alpha$, while increasing $p(c_{j})$ becomes $p\left(c_{j}\right)+\alpha /\left(n-1\right)$; (2) subtracting the constant α gives $p(c_{i})-\alpha$, while increasing $p(c_{j})$ gives $p\left(c_{j}\right)+\alpha /\left(1-p\left(c_{i}\right)\right)p\left(c_{j}\right)$; (3) press $p(c_{i})$ by the ratio of β to $\beta p(c_{i})$ while increasing $p(c_{j})$ gives $p\left(c_{j}\right)+\left(1-\beta \right)p\left(c_{i}\right)/\left(n-1\right)$; (4) $p(c_{i})$ reduced to β becomes $\beta p(c_{i})$, while increasing $p(c_{j})$ becomes $p\left(c_{j}\right)+\left(1-\beta \right)p\left(c_{i}\right)/\left(1-p\left(c_{i}\right)\right)p\left(c_{j}\right)$; (5) reduce $p(c_{i})$ to $1-\gamma (1-p_{i})$, while increasing $p(c_{j})$ to $\gamma p(c_{j})$.

## 4. Experiment and Discussion

### 4.1. Experiment Setup

To verify the effectiveness of the LPG–PCFG model, we conducted model implementation, training, and related comparative experiments on the rockyou [66] public dataset.

The implementation environment was the Ubuntu 16.04.6 LTS operating system; the programming language was python 3.6; the central processing unit used for program running was an Intel(R) Xeon(R) Silver 4116, with the main frequency being 2.10 GHz and 12 cores and 128 GB of memory.

The algorithm of the LPG–PCFG model in the training stage is shown in Algorithm 1. When the initial training stage was completed, the degenerate stage needed to be performed. The algorithm used in the related experiments in the degeneration stage of the LPG–PCFG model is shown in Algorithm 2. It should be noted that the speed affecting the model included the degeneration times, rules, range, and rate. The degeneration rules comprised the above five rules, and the degeneration range included password structures and different types of variables. The degeneration rule and range significantly affected model performance, and we explore them here in-depth. Finally, for the degeneration LPG–PCFG model, the random sampling algorithm used in the generation stage is shown in Algorithm 3.
**Algorithm 1:** LPG–PCFG model training algorithm.1 Set the initial password structure statistics as $T_{ps}$2 Set the numbers statistics as $T_{d}$3 Set the special characters statistics as $T_{s}$4  Set the letters n-gram statistics as $T_{l}$5  **for** password sample *x* in training set:6   Pause the structures of *x* and obtain $ps\;=\;v_{1}v_{2}v_{3}\cdots v_{n}$7   **for**
*v* in ps:8    **if**
*v* is number:9     Update $T_{d}$10    **end if**11    **if**
*v* is special characters:12     Update $T_{s}$13    **end if**14    **if**
*v* is letter:15     Update n-gram table16     Update $T_{l}$17    **end if**18   **end for**19   Update $T_{sp}$20  **end for**


**Algorithm 2:** LPG–PCFG model degeneration algorithm.1 Set degeneration rule as DR, degeneration times as $N_{D}$2  **for**
i=1:$N_{D}$:3   Generate a password *x* through random sampling4   Pause the structures of *x* and obtain $ps\;=\;v_{1}v_{2}v_{3}\cdots v_{n}$5   Randomly select a part from $ps,v_{s},v_{d},v_{l}$ to degenerate the probability6   **if** Selected and the changed part is a number:7    Change $T_{n}$ according to DR8   **end if**9   **if** The selected and the changed part is a letter:10    Randomly select an n-gram part to change11    Change $T_{l}$ according to DR12   **end if**13   **if** The selected and the changed part is a special character:14    Change $T_{s}$ according to DR15   **end if**16   **if** Selected and the changed part is structure:17    Change $T_{ps}$ according to DR18   **end if**19  **end for**


**Algorithm 3:** LPG–PCFG model generation algorithm.1 Set generation numbers as $N_{G}$2  **for**
i=1:$N_{G}$:3   Set *x* as an empty string4   Randomly select a password structure from $D_{ps}$5   **for**
*v* in ps:6    **if**
*v* is a number:7     Randomly select a number string form $T_{d}$8     Concat number string into *x*9    **end if**10    **if**
*v* is a letter:11     Continuously sample random letters according to the n-gram table $T_{l}$12     Concat letters into *x*13    **end if**14    **if**
*v* is a special character:15     Randomly select a special character string form $T_{s}$16     Concat special characters string into *x*17    **end if**18   **end for**19   *x* is a generated password20  **end for**


### 4.2. Effect of Parameter

First, in the LPG–PCFG model, the training set of the rockyou public dataset is used to complete model training and obtain the modeling distribution $D_{ori}$. Since the degeneration rule, range, rate, and times affect the distribution $D_{deg}$, we used the control variable method to examine, in turn, the influence of the above factors in the degeneration stage. To reasonably compare the performance of various degeneration distributions, a certain number of passwords in the experiment were first generated. When examining the hits of low-probability passwords, we counted the number of password hits in a test set in the range of $\left(10^{-12},10^{-9}\right)$.

#### 4.2.1. Effect of Degeneration Rate

For the five specific degeneration rules, we uniformly limited the probability adjustment range to the structure parts: letters, numbers, and special characters. The number of degeneration times was selected as $10^{6}$; the number of generated passwords was $10^{7}$; the adjustment rate was a gradient in an order of magnitude setup. The degeneration rates in Rules 1 and 2 were 0.99, 0.999, 0.9999, 0.99999, 0.999999, 0.9999999, 0.99999999; the degeneration rates in Rules 3 and 4 were $10^{-2},10^{-3},10^{-4},10^{-5},10^{-6},10^{-7},10^{-8}$; the degeneration rate of Rule 5 was 1.01, 1.001, 1.0001, 1.00001, 1.000001, 1.0000001, 1.00000001. The number of low-probability passwords hits in the test set is shown in Table 2, Table 3 and Table 4.

The experimental results show that the degeneration rate has a very significant effect on the model performance of LPG–PCFG. For example, in Rule 2, the degeneration rate of 0.99 and 0.9999999 produced an order of magnitude difference in the number of hits. For different degeneration rules, under the condition of $10^{6}$ degenerations, the relationship between the rate parameter and the number of low-probability password hits was also different. In Rules 1–3, with an increasing degeneration rate, the number of hits increased gradually and tended to be stable; in Rules 4 and 5, the number of hits increased first and then decreased, which achieved the best performance at $10^{-6}$ and 1.000001, respectively. Overall, when the degeneration rate was larger, the number of hits was generally lower, and when the degeneration rate was lower, LPG–PCFG tended to have a better performance. However, a too-low degeneration rate may not have generated a high hit count.

#### 4.2.2. Effect of Degeneration Range

Next, we discuss the effect of the degeneration range on model performance. In the LPG–PCFG model, the four parts—password structure, letters, numbers, and special characters (respectively, denoted as *p*, *l*, *n*, *s*)—are mutually independent in password probability calculations. These four parts also have different ranges of expression. Intuitively, the password structure has the most comprehensive expression range; for the letters and numbers, it is relatively weak; special characters usually have the lowest, so we set up multiple degeneration ranges. To perform a more detailed analysis of the degeneration effect, we also considered the influence of the degeneration rate and fixed the number of degeneration at $10^{6}$. The number of password generations was still set to $10^{7}$. The experimental results under Degeneration Rules 4 and 5 are shown in Table 5 and Table 6, respectively.

The experimental results showed that defining different degeneration ranges significantly affected model performance. When the range included the four parts, plns, the number of low-probability password hits varied with the degeneration rate and reached the maximum number of hits at a specific rate. When the degeneration range did not include the password structure (lns, ns, or *l*), the number of hits remained almost unchanged and relatively stable. When the degeneration range was *p* or plns, the changing trends were consistent, and both exhibited a significant increase in hits.

From the analysis of the above results, it could be seen that modifying the distribution of the password structure obtained a better degeneration distribution compared with letters, numbers, and special characters. In this regard, we believe that the structure distributions of high-probability and low-probability passwords are quite different. When the structural part of the passwords has degenerated, more patterns corresponding to low-probability passwords appear, so the random sampling can generate more low-probability passwords.

#### 4.2.3. Effect of Degeneration Times

Now, we discuss the effect of the number of degenerations. Section 4.2.2 showed that password structure is the optimal choice for the adjustment range, so in this section, we only considered adjusting the degenerate password structure. Since the adjustment rate significantly influences the number of hits, it was still set to the gradient configuration of Section 4.2.1 and Section 4.2.2. The number of degenerations was set to $10^{2}$, $10^{3}$, $10^{4}$, $10^{5}$, and $10^{6}$, respectively. The number of generated passwords remained set at $10^{7}$. The experimental results according to Degeneration Rules 4 and 5 are shown in Table 7 and Table 8.

It can be seen from the results that the impact of the number of degenerations on low-probability password hits was complex. Overall, for any number of degenerations, the number of hits still varied with the degeneration rate, and the highest number of hits was obtained at a specific degeneration rate. Second, for any degeneration rate, as the number of degenerations increased, the number of hits increased overall. However, the above two trends do not strictly conform to all experimental data. They may also violate phenomena under certain conditions, such as when the degeneration rate in Rule 5 is 1.00000001 and the degeneration number is $10^{4}$.

For the above experimental phenomena, we believe that the features of low-probability passwords are relatively insignificant enough, so the LPG–PCFG model often needs slow- and high-frequency adjustments when obtaining the degenerate distribution. Otherwise, the password features are easily lost. Higher degeneration times and lower degeneration rates enable fine-tuned distribution adjustments, while lower degeneration times may make the adjustment insufficient for a good degeneration distribution.

#### 4.2.4. Effect of Degeneration Rule

Based on the above experiments, we discuss how the degeneration rules affected the hits of low-probability passwords. We comprehensively considered three factors: degeneration rate, range, and times. The selection of the degeneration rate is shown in Section 4.2.1, the choice of degeneration range in Section 4.2.2, and the selection of degeneration times in Section 4.2.3. Under the fixed condition of $10^{7}$-times password generation, the optimal model under the five degeneration rules was screened. The results are shown in Table 9.

The experimental results showed that different degenerate rules will generate different numbers of low-probability password hits when generated $10^{7}$ times. Rules 1 and 3 can hit about 1600 passwords, while Rules 2, 4, and 5 can hit about 1600–2200. When the optimal performance of Rules 1 and 2 was obtained, the degeneration rate was 0.999999, and the degeneration times were $10^{5}$; the degeneration rate corresponding to the optimal models of Rules 3 and 4 was $10^{-7}$, and the degeneration times were both $10^{6}$. In addition, the degeneration ranges corresponding to the above five rules were all *p*, which is only the modified password structure.

### 4.3. Model Performance Comparison

**Figure 6:**
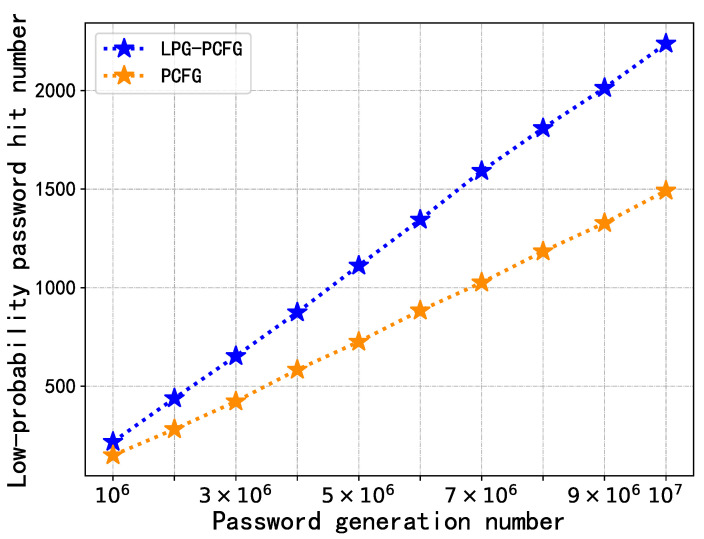
Performance comparison between LPG–PCFG and PCFG models (generated $10^{7}$ times).

**Figure 7:**
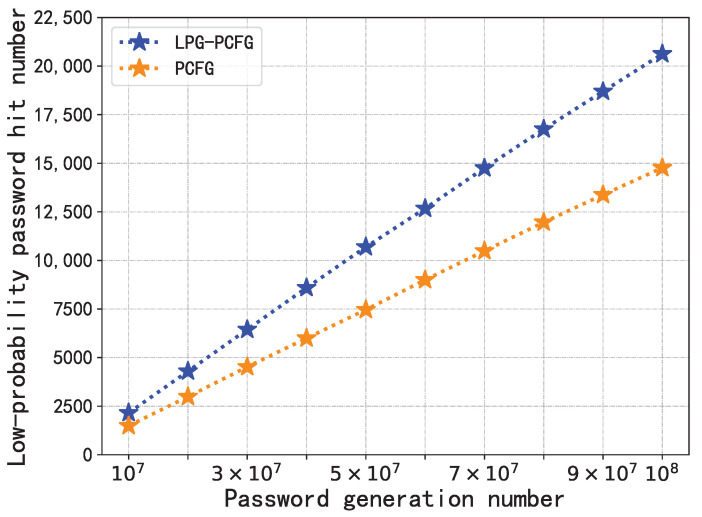
Performance comparison between LPG–PCFG and PCFG models (generated $10^{8}$ times).

Finally, we compared the performance of the LPG–PCFG model with the PCFG model. According to the conclusion of Section 4.2.4, the LPG–PCFG model with the best performance was selected according to Degeneration Rule 2; the range was *p*; the times were $10^{5}$; the rate was 0.999999. For a fine comparison of the performance of the modeling and degeneration distributions, the number of generated passwords was set to $10^{7}$ and $10^{8}$, respectively, and the number of hits to low-probability passwords is shown in Figure 6 and Figure 7.

The results showed that below $10^{7}$ generations, LPG–PCFG can hit 2238 low-probability passwords, while PCFG hit 1488, a relative increase of 50.4%; under generating $10^{8}$ times, LPG–PCFG can hit 21,208 passwords, while PCFG hit 14,932, a relative increase of 42.0%. It can be seen from the curve that LPG–PCFG can always hit more low-probability passwords than PCFG regardless of the number generated. The difference in the number of hits between the two will increase with the number of generated passwords.

To sum up, on the basis of PCFG, LPG–PCFG finally obtained a satisfactory degeneration distribution by finely controlling the degeneration rate, range, times, and rules. The degenerate distribution limits high-probability passwords and significantly increases the probability value of low-probability passwords. At this time, random sampling in the degenerate distribution found a larger number of low-probability passwords. The results fully demonstrated the effectiveness of the LPG–PCFG model.

However, this algorithm could not effectively improve the low-probability password hit capability of the LPG–PCFG model in all cases. Next, we discuss the different cases of model failure. The degradation rate represents the range of each degradation operation. The results showed that, in most cases, the degradation rate was too large or too small, limiting the model’s low-probability password generation ability. We believe there were two reasons when the degradation rate was too large. First, the probability distribution of the model was excessively degraded, resulting in a password with a lower probability that also had a similar probability to the password in the target interval, thus diluting the occurrence probability of the password in the low probability interval. Second, excessive changes caused by a single degradation may have damaged the modeling of password semantic features, resulting in model failure. In the research on the degradation range, we found that it was the most effective for password structure, but the worst for numbers and special characters. We believe that this phenomenon occurred mainly because the password structure part contained more semantic information; the combination of special symbols and specific numbers had less semantic information, so the effect of degenerate directional numbers and special characters was not good. In the degradation times, the failure situation was similar to the reason for the degradation rate. Insufficient times prevented an improvement in the password probability of the target interval. At the same time, too many degradation times led to the probability of excessive degradation and password dilution outside the target interval, which possibly impaired the modeling of semantic features. In the research on degenerate rules, Rules 1 and 3 did not work well, and the difference between these rules and others was that these two rules would even increase the probability of missing passwords. In comparison, the three different rules made passwords with relatively high probability boost relatively higher probability. We believe that the average increase in low-probability passwords may have caused password probability outside the target range to increase too quickly, thus diluting the probability of the occurrence of passwords in the target range.

## 5. Conclusions

The trained password-generation model completed the modeling of the password probability distribution. At this time, whether based on the random sampling method or enumeration method, high-probability passwords were straightforward to generate, while many low-probability passwords were not easily generated, resulting in insufficient coverage of low-probability-interval passwords. We analyzed the three aspects of password distribution, sampling method, and model design and proposed a degenerate distribution suitable for dealing with low-probability password generation. Based on the PCFG model, but with finer granularity, we presented the low-probability password-generation model LPG–PCFG based on a degenerate distribution. By finely tuning multiple factors such as degeneration rate, range, times, and rules, we obtained the optimal LPG–PCFG.

The LPG–PCFG model has the following advantages:

1. Compared with neural-network-based password generation, the LPG–PCFG model had high efficiency and low resource consumption in both the training and generation stages.
2. Compared with a state-of-the-art PCFG model based on statistical machine learning, LPG–PCFG had a significantly improved low-probability password generation. After $10^{7}$ generations, the number of hits increased by 50.4%, and after $10^{8}$ generations, LPG–PCFG had a relative improvement of 42.0%.
3. The degradation algorithm proposed in this paper is interpretable. We mathematically proved that in continuous degradation, the model’s evaluation of the password distribution gradually approached a uniform distribution, so the chances of finding a probabilistic password will gradually increase.

This paper proposed a degeneration algorithm to generate low-probability passwords in specific intervals that proved to be effective in a PCFG model. In password generation, neural networks show excellent performance; however, numerous learnable parameters usually lead to a certain degree of overfitting, which means that neural network model generalization is poor. In the following work, we will focus on applying this algorithm to solving the problems of overfitting and insufficient generalization in models of neural network password generation.

## Figures and Tables

**Table 1:** Five probability modification rules of degeneration distribution.

Rule	Adjust $p(x^{+})$	Adjust $p(x^{-})$
Rule 1	$p(x^{+})-\alpha$	$p\left(x\right)+\alpha /\left(N_{s}-1\right)$
Rule 2	$p(x^{+})-\alpha$	$p\left(x^{-}\right)+\alpha /\left(1-p\left(x^{+}\right)\right)p{(}^{-})$
Rule 3	$\beta p(x^{+})$	$p\left(x^{-}\right)+\left(1-\beta \right)p\left(x^{+}\right)\left(1-p\left(x^{+}\right)\right)p\left(x^{-}\right)$
Rule 4	$\beta p(x^{+})$	$p{\left(x^{-}\right)}_{(}1-\beta )p\left(x^{+}\right)\left(1-p\left(x^{+}\right)\right)p\left(x^{-}\right)$
Rule 5	$1-\gamma (1-p\left(x^{+}\right))$	$\gamma p(x^{-})$

**Table 2:** Effect of degeneration rate on low-probability password hits (Rules 1 and 2).

Degeneration Rate	0.99	0.999	0.9999	0.99999	0.999999	0.999999999	0.999999999
Rule 1	1587	1209	1447	1489	1532	1510	1492
Rule 2	510	1169	1436	1765	2011	1869	1580

**Table 3:** Effect of degeneration rate on low-probability password hits (Rules 3 and 4).

Degeneration Rate	$10^{-2}$	$10^{-3}$	$10^{-4}$	$10^{-5}$	$10^{-6}$	$10^{-7}$	$10^{-8}$
Rule 3	587	1209	1447	1489	1532	1510	1492
Rule 4	510	1169	1436	1765	2011	1869	1580

**Table 4:** Effect of degeneration rate on low-probability password hits (Rule 5).

Degeneration Rate	1.00000001	1.0000001	1.000001	1.00001	1.0001	1.001	1.01
Rule 5	1648	1815	1887	1653	1757	1113	992

**Table 5:** Effect of degeneration range on low-probability password hits (Rule 4).

Degeneration Range	$10^{-2}$	$10^{-3}$	$10^{-4}$	$10^{-5}$	$10^{-6}$	$10^{-7}$	$10^{-8}$
*plns*	510	1169	1436	1765	2011	1869	1580
*p*	1121	1350	1858	2061	2129	2218	1484
*lns*	1496	1542	1553	1579	1596	1520	1604
*ns*	1494	1516	1537	1546	1572	1604	1549
*l*	1512	1521	1551	1589	1592	1620	1533

**Table 6:** Effect of degeneration range on low-probability password hits (Rule 5).

Degeneration Range	1.00000001	1.0000001	1.000001	1.00001	1.0001	1.001	1.01
*plns*	1648	1815	1887	1653	1757	1113	992
*p*	1895	2301	2170	2081	2023	1177	1299
*lns*	1429	1545	1533	1628	1533	1487	1512
*ns*	1508	1489	1602	1568	1448	1608	1567
*l*	1532	1575	1575	1537	1578	1498	1552

**Table 7:** Effect of degeneration times on low-probability password hits (Rule 4).

Degeneration Times	$10^{-2}$	$10^{-3}$	$10^{-4}$	$10^{-5}$	$10^{-6}$	$10^{-7}$	$10^{-8}$
$10^{2}$	1243	1205	1965	2015	1863	1722	1880
$10^{3}$	1456	1432	1922	2119	2083	1675	1484
$10^{4}$	1546	1349	1968	2160	1995	2109	1949
$10^{5}$	1671	1544	1760	2001	2187	1941	1541
$10^{6}$	1121	1350	1858	2061	2129	2218	1989

**Table 8:** Effect of degeneration times on low-probability password hits (Rule 5).

Degeneration Times	1.00000001	1.0000001	1.000001	1.00001	1.0001	1.001	1.01
$10^{2}$	1599	1637	1620	1571	1429	1450	1501
$10^{3}$	1922	1934	2003	1710	1814	1277	1439
$10^{4}$	2230	2167	2169	1790	1968	1881	1514
$10^{5}$	2026	2015	2169	2054	1949	2150	2019
$10^{6}$	1895	2301	2170	2081	2023	1177	1299

**Table 9:** Maximum number of low-probability password hits with different degeneration rules.

Degeneration Rules	Degeneration Speed	Degeneration Range	Degeneration Times	Hit
Rule 1	0.999999	*p*	$10^{5}$	1624
Rule 2	0.999999	*p*	$10^{5}$	2238
Rule 3	$10^{-7}$	*p*	$10^{6}$	1688
Rule 4	$10^{-7}$	*p*	$10^{6}$	2218
Rule 5	1.0000001	*p*	$10^{6}$	2301

## Data Availability

The dataset we used in this study is the Rockyou dataset, and it is openly available in https://dx.doi.org/10.21227/gzcg-yc14 (accessed on 10 May 2022).

## References

[1] Lee P.Y., Choong Y.Y. (2015). Human generated passwords—the impacts of password requirements and presentation styles. Proceedings of the International Conference on Human Aspects of Information Security, Privacy, and Trust.

[2] Garrett K., Talluri S.R., Roy S. (2015). On vulnerability analysis of several password authentication protocols. Innov. Syst. Softw. Eng..

[3] Li Z., He W., Akhawe D., Song D. The {Emperor’s} New Password Manager: Security Analysis of Web-based Password Managers. Proceedings of the 23rd USENIX Security Symposium (USENIX Security 14).

[4] Mannuela I., Putri J., Anggreainy M.S. (2021). Level of Password Vulnerability. Proceedings of the 2021 1st International Conference on Computer Science and Artificial Intelligence (ICCSAI).

[5] Zhao R., Yue C., Sun K. (2013). Vulnerability and risk analysis of two commercial browser and cloud based password managers. ASE Sci. J..

[6] Katz J., Ostrovsky R., Yung M. (2001). Efficient password-authenticated key exchange using human-memorable passwords. Proceedings of the International Conference on the Theory and Applications of Cryptographic Techniques.

[7] Park S.B., Kang M.S., Lee S.J. (2003). User authentication protocol based on human memorable password and using ECC. Proceedings of the International Conference on Grid and Cooperative Computing.

[8] Arroyabe I.F.D., de Arroyabe J.C.F. (2021). The severity and effects of Cyber-breaches in SMEs: A machine learning approach. Enterp. Inf. Syst..

[9] Singh N., Krishnaswamy V., Zhang J.Z. (2022). Intellectual structure of cybersecurity research in enterprise information systems. Enterp. Inf. Syst..

[10] Nathan M. (2020). Credential stuffing: New tools and stolen data drive continued attacks. Comput. Fraud Secur..

[11] Lin C.W., Tsai C.S., Hwang M.S. (2006). A new strong-password authentication scheme using one-way hash functions. J. Comput. Syst. Sci. Int..

[12] Juels A., Rivest R.L. Honeywords: Making password-cracking detectable. Proceedings of the 2013 ACM SIGSAC Conference on Computer & Communications Security.

[13] Jeong J., Woo D., Cha Y. (2019). Enhancement of website password security by using access log-based salt. Proceedings of the 2019 International Conference on Systems of Collaboration Big Data, Internet of Things & Security (SysCoBIoTS).

[14] Gauravaram P. (2012). Security Analysis of salt|| password Hashes. Proceedings of the 2012 International Conference on Advanced Computer Science Applications and Technologies (ACSAT).

[15] Boonkrong S., Somboonpattanakit C. (2016). Dynamic salt generation and placement for secure password storing. IAENG Int. J. Comput. Sci..

[16] Narayanan A., Shmatikov V. Fast dictionary attacks on passwords using time–space tradeoff. Proceedings of the 12th ACM Conference on Computer and Communications Security.

[17] Ah Kioon M.C., Wang Z.S., Deb Das S. (2013). Security analysis of MD5 algorithm in password storage. Applied Mechanics and Materials.

[18] Marechal S. (2008). Advances in password cracking. J. Comput. Virol..

[19] Liu P., Li S., Ding Q. (2018). An energy-efficient accelerator based on hybrid CPU-FPGA devices for password recovery. IEEE Trans. Comput..

[20] Zhang Z., Liu P., Wang W., Li S., Wang P., Jiang Y. (2020). High-Performance Password Recovery Hardware Going From GPU to Hybrid CPU-FPGA Platform. IEEE Consum. Electron. Mag..

[21] Tirado E., Turpin B., Beltz C., Roshon P., Judge R., Gagneja K. (2018). A new distributed brute-force password cracking technique. Proceedings of the International Conference on Future Network Systems and Security.

[22] Hranickỳ R., Holkovič M., Matoušek P. (2016). On efficiency of distributed password recovery. J. Digit. Forensics Secur. Law.

[23] Chou H.C., Lee H.C., Yu H.J., Lai F.P., Huang K.H., Hsueh C.W. (2013). Password cracking based on learned patterns from disclosed passwords. Int. J. Innov. Comput. Inf. Control.

[24] Pasquini D., Gangwal A., Ateniese G., Bernaschi M., Conti M. (2021). Improving password guessing via representation learning. Proceedings of the 2021 IEEE Symposium on Security and Privacy (SP).

[25] Deng G., Yu X., Guo H. (2019). Efficient password guessing based on a password segmentation approach. Proceedings of the 2019 IEEE Global Communications Conference (GLOBECOM).

[26] Vaithyasubramanian S., Christy A., Saravanan D. (2014). An analysis of Markov password against brute force attack for effective web applications. Appl. Math. Sci..

[27] Jablon D.P. (1997). Extended password key exchange protocols immune to dictionary attack. Proceedings of the IEEE 6th Workshop on Enabling Technologies: Infrastructure for Collaborative Enterprises.

[28] Chakrabarti S., Singhal M. (2007). Password-based authentication: Preventing dictionary attacks. Computer.

[29] Shay R., Komanduri S., Kelley P.G., Leon P.G., Mazurek M.L., Bauer L., Christin N., Cranor L.F. Encountering stronger password requirements: User attitudes and behaviors. Proceedings of the Sixth Symposium on Usable Privacy and Security.

[30] Weir M., Aggarwal S., De Medeiros B., Glodek B. (2009). Password cracking using probabilistic context-free grammars. Proceedings of the 2009 30th IEEE Symposium on Security and Privacy.

[31] Melicher W., Ur B., Segreti S.M., Komanduri S., Bauer L., Christin N., Cranor L.F. Fast, lean, and accurate: Modeling password guessability using neural networks. Proceedings of the 25th {USENIX} Security Symposium ({USENIX} Security 16).

[32] Hitaj B., Gasti P., Ateniese G., Perez-Cruz F. (2019). Passgan: A deep learning approach for password guessing. Proceedings of the International Conference on Applied Cryptography and Network Security.

[33] Hranickỳ R., Lištiak F., Mikuš D., Ryšavỳ O. (2019). On practical aspects of pcfg password cracking. Proceedings of the IFIP Annual Conference on Data and Applications Security and Privacy.

[34] Saputra R., Noranita B. (2019). Analysis of GPGPU-Based Brute-Force and Dictionary Attack on SHA-1 Password Hash. Proceedings of the 2019 3rd International Conference on Informatics and Computational Sciences (ICICoS).

[35] Nguyen D.H., Nguyen T.T., Duong T.N., Pham P.H. Cryptanalysis of MD5 on GPU Cluster. Proceedings of the International Conference on Information Security and Artificial Intelligence.

[36] Mentens N., Batina L., Preneel B., Verbauwhede I. (2006). Time-memory trade-off attack on FPGA platforms: UNIX password cracking. Proceedings of the International Workshop on Applied Reconfigurable Computing.

[37] Bošnjak L., Brumen B. (2019). Rejecting the death of passwords: Advice for the future. Comput. Sci. Inf. Syst..

[38] Wang D., Wang P. (2015). Offline dictionary attack on password authentication schemes using smart cards. Information Security.

[39] (2017). Hashcat Advanced Password Recovery. https://hashcat.net/wiki/.

[40] (2017). John the Ripper Password Cracker. http://www.openwall.com/john/.

[41] Limpanuparb T. (2004). The Enhancement of Password Security System Using Key Stroke Verification. NECTEC Tech. J..

[42] Houshmand S., Aggarwal S., Flood R. (2015). Next gen PCFG password cracking. IEEE Trans. Inf. Forensics Secur..

[43] Veras R., Collins C., Thorpe J. (2021). A Large-Scale Analysis of the Semantic Password Model and Linguistic Patterns in Passwords. ACM Trans. Priv. Secur. (TOPS).

[44] Li Y., Wang H., Sun K. A study of personal information in human-chosen passwords and its security implications. Proceedings of the IEEE INFOCOM 2016—IEEE Conference on Computer Communications.

[45] Zhang Y., Xian H., Yu A. (2020). CSNN: Password guessing method based on Chinese syllables and neural network. Peer Netw. Appl..

[46] Han W., Xu M., Zhang J., Wang C., Zhang K., Wang X.S. (2020). TransPCFG: Transferring the grammars from short passwords to guess long passwords effectively. IEEE Trans. Inf. Forensics Secur..

[47] Tansey W. (2011). Improved Models for Password Guessing. https://www.semanticscholar.org/paper/Improved-Models-for-Password-Guessing-Tansey/3451ac7f102da12e1197c681b77d368ba3b19ac9.

[48] Dürmuth M., Angelstorf F., Castelluccia C., Perito D., Chaabane A. OMEN: Faster Password Guessing Using an Ordered Markov Enumerator. Proceedings of the International Symposium on Engineering Secure Software & Systems.

[49] Guo X., Liu Y., Tan K., Mao W., Jin M., Lu H. (2021). Dynamic Markov Model: Password Guessing Using Probability Adjustment Method. Appl. Sci..

[50] Bodkhe U., Chaklasiya J., Shah P., Tanwar S., Vora M. (2020). Markov model for password attack prevention. Proceedings of the First International Conference on Computing, Communications, and Cyber-Security (IC4S 2019).

[51] Linghu Y., Li X., Zhang Z. (2019). Deep Learning vs. Traditional Probabilistic Models: Case Study on Short Inputs for Password Guessing. Proceedings of the International Conference on Algorithms and Architectures for Parallel Processing.

[52] Sutskever I., Martens J., Hinton G. (2011). Generating Text with Recurrent Neural Networks.

[53] Xu L., Ge C., Qiu W., Huang Z., Lian H. Password Guessing Based on LSTM Recurrent Neural Networks. Proceedings of the IEEE International Conference on Computational Science & Engineering.

[54] Nanjun T., Huaxiang L.U., Min J., Junbin Y.E., Zhiyuan L.I. (2018). PG-RNN: A password-guessing model based on recurrent neural networks. CAAI Trans. Intell. Syst..

[55] Goodfellow I.J., Pouget-Abadie J., Mirza M., Xu B., Warde-Farley D., Ozair S., Courville A., Bengio Y. (2014). Generative Adversarial Networks. Adv. Neural Inf. Process. Syst..

[56] Kingma D.P., Dhariwal P. (2018). Glow: Generative flow with invertible 1x1 convolutions. arXiv.

[57] Salimans T., Karpathy A., Chen X., Kingma D.P. (2017). Pixelcnn++: Improving the pixelcnn with discretized logistic mixture likelihood and other modifications. arXiv.

[58] Croce D., Castellucci G., Basili R. GAN-BERT: Generative adversarial learning for robust text classification with a bunch of labeled examples. Proceedings of the 58th Annual Meeting of the Association for Computational Linguistics.

[59] Nam S., Jeon S., Moon J. (2020). Generating Optimized Guessing Candidates toward Better Password Cracking from Multi-Dictionaries Using Relativistic GAN. Appl. Sci..

[60] Nam S., Jeon S., Kim H., Moon J. (2020). Recurrent gans password cracker for iot password security enhancement. Sensors.

[61] Guo X., Liu Y., Tan K., Jin M., Lu H. (2021). PGGAN: Improve Password Cover Rate Using the Controller. Journal of Physics: Conference Series.

[62] Srivastava A., Valkov L., Russell C., Gutmann M.U., Sutton C. (2017). Veegan: Reducing mode collapse in gans using implicit variational learning. arXiv.

[63] LeCun Y., Bengio Y., Hinton G. (2015). Deep learning. Nature.

[64] Zipf G.K. (2016). Human Behavior and the Principle of Least Effort: An Introduction to Human Ecology.

[65] Wang D., Cheng H., Wang P., Huang X., Jian G. (2017). Zipf’s Law in Passwords. IEEE Trans. Inf. Forensics Secur..

[66] (2017). Skullsecurity. RockYou. https://wiki.skullsecurity.org/Passwords.

